# GPR124 regulates murine brain embryonic angiogenesis and BBB formation by an intracellular domain-independent mechanism

**DOI:** 10.1242/dev.202794

**Published:** 2024-06-17

**Authors:** Kanako Yuki, Mario Vallon, Jie Ding, Cara C. Rada, Alan T. Tang, José G. Vilches-Moure, Aaron K. McCormick, Maria F. Henao Echeverri, Samira Alwahabi, Barbara M. Braunger, Süleyman Ergün, Mark L. Kahn, Calvin J. Kuo

**Affiliations:** ^1^ Department of Medicine, Division of Hematology, Stanford University School of Medicine, Stanford, CA 94305, USA; ^2^ Institute of Anatomy and Cell Biology, Julius-Maximilians-University Wuerzburg, 97070 Wuerzburg, Germany; ^3^ Department of Medicine and Cardiovascular Institute, University of Pennsylvania, Philadelphia, PA 19104, USA; ^4^ Department of Comparative Medicine, Stanford University School of Medicine, Stanford, CA 94305, USA

**Keywords:** GPR124, WNT signaling, CNS angiogenesis, Blood-brain barrier, Endothelial cells

## Abstract

The GPR124/RECK/WNT7 pathway is an essential regulator of CNS angiogenesis and blood-brain barrier (BBB) function. GPR124, a brain endothelial adhesion seven-pass transmembrane protein, associates with RECK, which binds and stabilizes newly synthesized WNT7 that is transferred to frizzled (FZD) to initiate canonical β-catenin signaling. GPR124 remains enigmatic: although its extracellular domain (ECD) is essential, the poorly conserved intracellular domain (ICD) appears to be variably required in mammals versus zebrafish, potentially via adaptor protein bridging of GPR124 and FZD ICDs. GPR124 ICD deletion impairs zebrafish angiogenesis, but paradoxically retains WNT7 signaling upon mammalian transfection. We thus investigated GPR124 ICD function using the mouse deletion mutant *Gpr124^ΔC^*. Despite inefficiently expressed GPR124^ΔC^ protein, *Gpr124^ΔC/ΔC^* mice could be born with normal cerebral cortex angiogenesis, in comparison with *Gpr124^−/−^* embryonic lethality, forebrain avascularity and hemorrhage. *Gpr124^ΔC/ΔC^* vascular phenotypes were restricted to sporadic ganglionic eminence angiogenic defects, attributable to impaired GPR124^ΔC^ protein expression. Furthermore, Gpr124^ΔC^ and the recombinant GPR124 ECD rescued WNT7 signaling in culture upon brain endothelial *Gpr124* knockdown. Thus, in mice, GPR124-regulated CNS forebrain angiogenesis and BBB function are exerted by ICD-independent functionality, extending the signaling mechanisms used by adhesion seven-pass transmembrane receptors.

## Teaser

**Summary:** Mouse GPR124 mutants lacking the intracellular domain exhibit normal developmental cerebral cortex angiogenesis and blood-brain barrier function.

## INTRODUCTION

The cerebrovasculature is characterized by possession of a blood-brain barrier (BBB), which is a tightly regulated interface comprising cooperating cellular, junctional and transporter elements, that controls the influx and efflux of biological substances between the brain parenchyma and blood. BBB dysfunction is associated with the progression of CNS diseases such as ischemic stroke, neurodegeneration and brain tumors (Rada et al., 2023; Wu et al., 2023). Endothelial canonical WNT/β-catenin signaling, specifically regulated by WNT7A/WNT7B, is essential for CNS angiogenesis and BBB formation (Stenman et al., 2008; Daneman et al., 2009).

General canonical WNT/β-catenin signaling is activated by the binding of WNT ligands to the receptor frizzled (FZD1-10), with subsequent heterodimerization of FZD and LRP5/6 co-receptors inducing stabilization of β-catenin, which interacts with TCF/LEF transcription factors and activates transcription of WNT target genes (Janda et al., 2017; Nusse and Clevers, 2017). The seven-pass transmembrane protein G-protein-coupled receptor 124 (GPR124) and GPI-anchored protein RECK are essential membrane co-activators that selectively amplify WNT7 signaling in concert with FZD and LRP in the CNS endothelium (Posokhova et al., 2015; Vanhollebeke et al., 2015; Cho et al., 2017; Vallon et al., 2018; Cho et al., 2019). Genetic knockout of *Gpr124* or *Reck*, or double knockout of *Wnt7a* and *Wnt7b*, all elicit common embryonic lethal CNS angiogenesis and hemorrhage phenotypes that are reversed by genetic WNT/β-catenin signaling activation (Oh et al., 2001; Stenman et al., 2008; Daneman et al., 2009; Kuhnert et al., 2010; Anderson et al., 2011; Cullen et al., 2011; Zhou and Nathans, 2014; de Almeida et al., 2015; Posokhova et al., 2015; Chang et al., 2017; Cho et al., 2017, 2019). GPR124 and canonical WNT/β-catenin signaling are required for BBB maintenance during pathological states such as stroke (Chang et al., 2017; Jean LeBlanc et al., 2019; Song et al., 2021; Hussain et al., 2022; Martin et al., 2022). RECK is a specific receptor that binds WNT7A and WNT7B through CK and CRD domains (Eubelen et al., 2018; Vallon et al., 2018; Cho et al., 2019), stabilizes newly secreted WNT7 into an active monomeric form and increases FZD8-WNT7 complex formation (Vallon et al., 2018). RECK also binds to GPR124 through RECK CK/EGF2 to GPR124 LRR/GAIN domains extracellularly to promote canonical WNT/β-catenin signaling (Cho et al., 2017; Eubelen et al., 2018; Vallon et al., 2018; Cho et al., 2019). In mice, mutation of the RECK CK domain induces severe CNS defects, suggesting that this motif regulates canonical WNT signaling activation (Cho et al., 2019).

In contrast to RECK, the molecular function of GPR124 is poorly understood. The BBB is conserved across vertebrates, in which conserved prominent canonical WNT signaling drives brain angiogenesis and BBB formation. Although GPR124- and RECK-dependent canonical WNT signaling regulates endothelial tip cell properties and is required for brain angiogenesis in zebrafish (Vanhollebeke et al., 2015; Eubelen et al., 2018), species-specific differences in the molecular mechanisms may exist. GPR124 extracellular domain (ECD) deletion diminishes WNT7 signaling activity in human, and in zebrafish GPR124 cell-based assays and zebrafish hindbrain angiogenesis models (Posokhova et al., 2015; Eubelen et al., 2018; Vallon et al., 2018). Both human and mouse, but not zebrafish, GPR124 ECD recombinant proteins are sufficient to induce WNT7 signaling (Vallon et al., 2018; America et al., 2022). The deletion of the zebrafish GPR124 intracellular domain (ICD) significantly impairs embryonic brain angiogenesis and WNT signaling activation in cell-based assays (Eubelen et al., 2018; America et al., 2022), whereas deletion of human or mouse GPR124 ICD only partially reduces WNT7 signaling (Posokhova et al., 2015; Vallon et al., 2018; America et al., 2022).

The GPR124 ICD is poorly conserved between zebrafish and mammals (<40%) whereas the mouse-human identity is ∼80% (America et al., 2022). The zebrafish GPR124 ICD recruits the scaffold/adaptor proteins Dishevelled, or DLG4 and MAGI3, via distinct DVL and ETTV PDZ-binding motifs, which act in a partially redundant manner during zebrafish brain angiogenesis. In contrast, mammalian GPR124 ICD does not bind DVL but does bind DLG1, and ETTV deletion retains partial signaling activity (Posokhova et al., 2015; America et al., 2022). GPR124 ICD mutants from human and mouse, but not zebrafish, exhibit intermediate rescue of brain angiogenesis in zebrafish *gpr124* morphant embryos (America et al., 2022). Thus, accumulating results indicate evolutionary differences regarding GPR124 ICD function in zebrafish versus mammalian cell culture systems, suggesting essentiality in the former, but not the latter (Vallon et al., 2018; America et al., 2022).

To directly explore GPR124 ICD function in mammals, we thus created a murine *Gpr124^ΔC^* allele lacking the C-terminal intracellular domain. Notably, *Gpr124^ΔC/ΔC^* mice could be born and did not phenocopy embryonic lethal hemorrhagic defects on brain angiogenesis and BBB phenotypes of *Gpr124*-null mice. This suggested that murine GPR124 can function in an ICD-independent manner, which was confirmed by signaling activity of the ECD.

## RESULTS

### GPR124 ICD deletion mice produce viable pups without brain hemorrhage

**Fig. 1.**
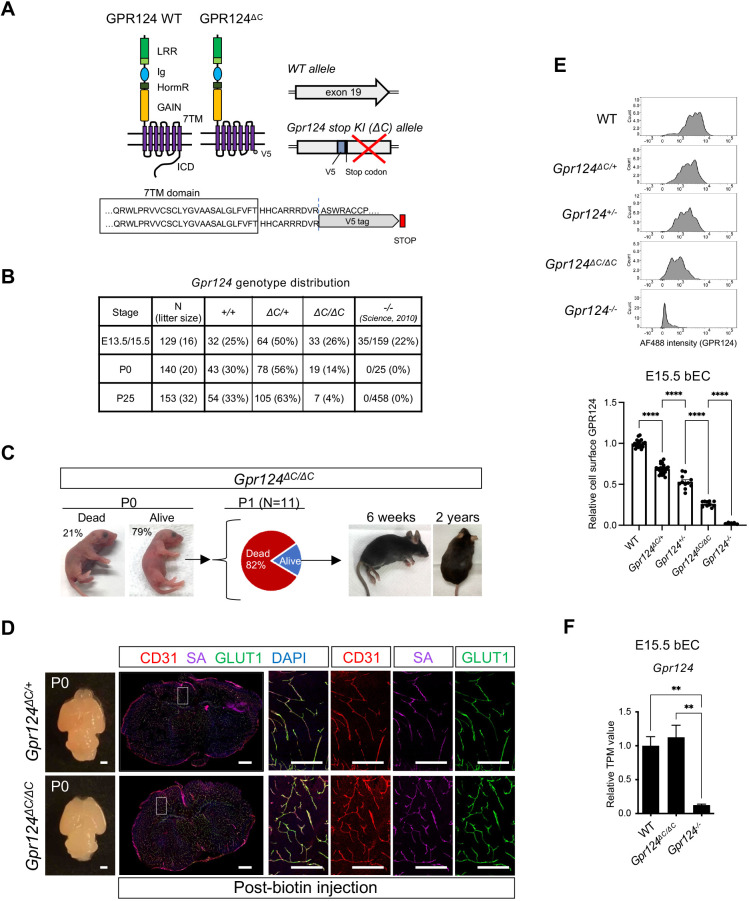
**GPR124 ICD deletion yields viable pups without brain hemorrhage.** (A) Schematic of Gpr124^ΔC^ depicting the extracellular domain (ECD), composed of LRR, Ig-like, HormR and GAIN, the transmembrane domain (7TM), and the intracellular domain (ICD). A V5 tag and stop codon were inserted before the ICD. (B) Genotype distribution of offspring from *Gpr124^ΔC/+^* heterozygous intercrosses. *P*-values were calculated using a chi-square test. **P<0.01, ***P<0.001. The data in the right column (*Gpr124^−/−^*) are taken from Kuhnert et al. (2010). (C) Survival of *Gpr124^ΔC/ΔC^* mice. Images of *Gpr124^ΔC/ΔC^* mice from postnatal (P0) to adult stages (6 weeks to 2 years) are depicted. Although *Gpr124^ΔC/ΔC^* mice were readily born (P0), substantial death occurred by P1 (*n*=11 analyzed). Mice that survived P1 then typically exhibited normal survival thereafter. Of note, 11 *Gpr124^ΔC/ΔC^* mice were used for tracking experiments and others were used for other experiments. Some litters checked at P25 were not checked at P0. (D) Dorsal bright-field view and immunofluorescence staining of cortex at P0 injected with sulfo-NHS-biotin. Fluorescent-conjugated streptavidin (SA, purple) was used to detect biotin. Glucose transporter GLUT1 (green), CD31 (red) and DAPI (blue). Scale bars: 1 mm for bright-field images; 500 μm for IF staining images. Boxed regions are shown at higher magnification and with single channels in the right-hand panels. Scale bars: 200 μm. (E) FACS histogram plots of cell-surface GPR124 detected using anti-GPR124 ECD antibody in E15.5 CD31+ brain endothelial cells from embryo forebrains of the indicated genotypes (top). The mean fluorescence intensity (MFI) was normalized to wild-type littermate controls (bottom). Wild type, *n*=21; *Gpr124^ΔC/+^*, *n*=25; *Gpr124^+/−^*, *n*=11; *Gpr124^ΔC/ΔC^*, *n*=10; *Gpr124^−/−^*, *n*=5. (F) *Gpr124* expression from RNA-seq of E15.5 FACS-purified brain EC in wild type, *Gpr124^ΔC/ΔC^* and *Gpr124^−/−^*. Wild type, *n*=4; *Gpr124^ΔC/ΔC^*, *n*=4; *Gpr124*^−/−^, *n*=4. Data are mean±s.e.m. Two-sided *P*-values were calculated using one-way ANOVA Tukey's multiple comparisons test. **P<0.01, ****P<0.0001.

GPR124 contains a large extracellular component consisting of a leucine-rich repeat (LRR) domain, an immunoglobulin (Ig) domain, a hormone receptor (HormR) domain and a putative GPCR autoproteolysis-inducing (GAIN) domain. In addition, GPR124 also has a seven-transmembrane domain (7TM) and C-terminal intracellular region (Fig. 1A). To investigate possible roles of the GPR124 ICD in CNS angiogenesis and canonical WNT/β-catenin signaling in mice, we deleted the cytoplasmic C-terminal tail by inserting a V5 tag and stop codon after Arg 1074, truncating the protein 10 amino acids downstream of transmembrane domain 7. This mouse allele was defined as *Gpr124^ΔC^* (Fig. 1A).

Global or conditional endothelial specific knockout of *Gpr124* or its co-receptor *Reck* all exhibit embryonic lethality accompanied with defects in CNS angiogenesis and BBB development, including severe hemorrhage around E13-E15 (Kuhnert et al., 2010; Anderson et al., 2011; Zhou and Nathans, 2014). In striking contrast, homozygous *Gpr124^ΔC/ΔC^* E13.5 and E15.5 knock-in embryos generated from *Gpr124^ΔC/+^* heterozygous intercrosses were viable and identified at close to expected Mendelian ratios (25%) (Fig. 1B). Furthermore, *Gpr124^ΔC/ΔC^* mice could be born and did not exhibit brain hemorrhage (Fig. 1B-D) but postnatal survival was less than the expected 25% through postnatal day 0 (P0) to young adulthood (P25) (Fig. 1B). 8 of 19 pups were used for the postnatal analysis. Tracking *Gpr124^ΔC/ΔC^* mice from P0 to adulthood, 9 of 11 pups were born but underwent postnatal death at P0 (Fig. 1C). Notably, *Gpr124^ΔC/ΔC^* mice that survived until P0 lived for more than 2 years (Fig. 1C).

Brain endothelial cells of *Gpr124^ΔC/ΔC^* embryos and adult mice were analyzed by flow cytometry, cell surface ELISA and immunoblotting to confirm protein expression. This revealed a 70% decrease in GPR124^ΔC^ cell surface protein expression and in total protein levels, as opposed to wild-type GPR124 (Fig. S1A,B). Notably, we observed a progressive decrease in brain endothelial GPR124 cell surface expression by FACS with a relative rank order of *Gpr124^+/+^* (wild type)>*Gpr124^ΔC/+^* > *Gpr124^+/−^* > *Gpr124^ΔC/ΔC^*>*Gpr124^−/−^* (Fig. 1E). In contrast, *Gpr124^ΔC^* mRNA was expressed similarly to wild-type *Gpr124* mRNA in embryos and adults (Fig. 1F and Fig. S1C), suggesting relative instability of GPR124^ΔC^ protein.

Global or endothelial specific *Gpr124* knockout exhibits BBB disruption, leading to severe brain hemorrhaging in embryos (Kuhnert et al., 2010; Anderson et al., 2011; Cullen et al., 2011; Zhou and Nathans, 2014). To assess the effect of GPR124 ICD deletion in postnatal BBB integrity, sulfo-NHS-biotin (443 Da) or sodium fluorescein (NaFl) (376 Da) small molecule tracers were intraperitoneally injected into P0 *Gpr124^+/+^* and *Gpr124^ΔC/ΔC^* pups. Sulfo-NHS-biotin detected by streptavidin colocalized with CD31+ endothelial cells but no sulfo-NHS-biotin/NaFl leakage into the brain parenchyma was observed in either *Gpr124^+/+^*, *Gpr124^ΔC/+^* or *Gpr124^ΔC/ΔC^* pups (Fig. 1D and Fig. S2A,B). Further staining of BBB markers, the tight junction proteins CLDN5 and ZO1 as well as the glucose transporter GLUT1, demonstrated normal vascularization with maintained BBB marker expression and colocalization in CD31+ vascular endothelial cells of *Gpr124^ΔC/ΔC^* brain (Fig. S2C). Previous reports have shown completely penetrant failure of secondary palate formation in *Gpr124^−/−^* knockout embryos (Anderson et al., 2011). Upon histological analysis at P0, including dead pups (wild type, *n*=2; *Gpr124^ΔC/+^*, *n*=4; *Gpr124^ΔC/ΔC^*, *n*=8 including alive, *n*=1, and dead, *n*=7), three out of eight *Gpr124^ΔC/ΔC^* mice exhibited a cleft palate (Fig. S3A,B), suggesting a mild phenotype versus *Gpr124^−/−^* null mice (Anderson et al., 2011). Overall, despite decreased expression of GPR124^ΔC^ protein, *Gpr124^ΔC/ΔC^* mice could survive and manifest normal BBB function, in strong contrast to embryonic lethal *Gpr124^−/−^* phenotypes.

### GPR124 ICD deletion does not recapitulate *Gpr124* knockout CNS defects

**Fig. 2.**
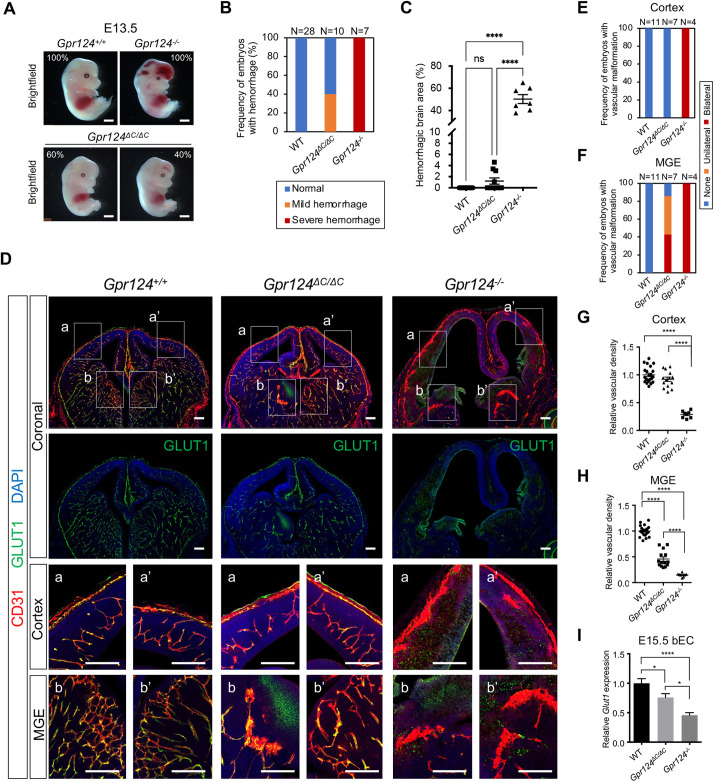
**GPR124 ICD deletion does not recapitulate *Gpr124^−/−^* CNS defects.** (A) Bright-field images of E13.5 embryos. Percentage indicates the frequency of representative phenotypes in the indicated embryo genotypes. Scale bar: 1 mm. (B) Frequency of embryos with forebrain hemorrhage at E13.5. (C) Relative hemorrhage size in whole brain at E13.5. Wild type, *n*= 28; *Gpr124^ΔC/ΔC^*, *n*=10; *Gpr124^−/−^*, *n*=7. (D) Immunofluorescent staining of forebrain coronal sections in E13.5 embryos. Boxed regions in the cortex (a and a′) and MGE (b and b′) are shown at higher magnification in the enlargements. Scale bars: 200 μm. (E,F) Frequency of embryos with glomeruloid malformations in cortex (E) and MGE (F). Embryos were categorized as having unilateral or bilateral malformations, or absence of malformations. Wild type, *n*=11; *Gpr124^ΔC/ΔC^*, *n*=7; *Gpr124^−/−^*, *n*=4. (G,H) Quantification of the relative vascular density in cortex (G) and MGE (H) for each genotype shown in D. CD31+ vasculature in both cortical hemispheres and MGE were quantified and normalized to wild-type littermate controls. WT, *n*=11; *Gpr124^ΔC/ΔC^*, *n*=7; *Gpr124^−/−^*, *n*=4. (I) Expression of *Glut1*, as assessed by RT-qPCR, in sorted CD31+ brain endothelial cells from E15.5 wild-type, *Gpr124^ΔC/ΔC^* and *Gpr124^−/−^* embryo forebrains*.* Each value was normalized to *Gapdh* and wild-type littermate controls. Wild type, *n*=8; *Gpr124^ΔC/ΔC^*, *n*=8; *Gpr124^−/−^*, *n*=6. Data are mean±s.e.m. Two-sided *P*-values were calculated using Tukey's multiple comparisons test. *P<0.05 and ****P<0.0001.

Next, we compared embryonic CNS angiogenesis in *Gpr124^ΔC/ΔC^* versus *Gpr124^−/−^* embryos. *Gpr124^−/−^* embryos exhibit severe hemorrhage in the forebrain and spinal cord (Kuhnert et al., 2010; Anderson et al., 2011; Cullen et al., 2011; Zhou and Nathans, 2014; Posokhova et al., 2015) whereas *Gpr124^ΔC/ΔC^* embryos manifested either absent or mild forebrain hemorrhage (Fig. 2A). Only 40% of *Gpr124^ΔC/ΔC^* embryos showed any degree of brain hemorrhage upon bright-field microscopy, which, if present at all, was significantly smaller than in *Gpr124^−/−^* (Fig. 2A-C). In wild-type mouse embryos, cortex vessels migrate and sprout from the perineural vascular plexus (PNVP) into the ventral forebrain and ganglionic eminences to form vascular networks (Fig. 2D, left column). As previously reported, *Gpr124^−/−^* embryos displayed severe vascular defects, with reduced angiogenic sprouting and migration, and glomeruloid malformations both in cortex and middle ganglionic eminences (MGE), accompanied by large ventricles (Fig. 2D right column; Fig. 2E-H) (Kuhnert et al., 2010; Anderson et al., 2011; Cullen et al., 2011; Zhou and Nathans, 2014; Posokhova et al., 2015). On the other hand, *Gpr124^ΔC/ΔC^* embryos exhibited normal telencephalon vascular patterning and density in cortex, similar to wild type (Fig. 2D middle column, a, a′, Fig. 2E,G). Spinal cord angiogenic deficits or hemorrhage were not observed in *Gpr124^ΔC/ΔC^* embryos, in marked contrast to *Gpr124^−/−^* embryos (Fig. S3C, Fig. 2A) (Kuhnert et al., 2010; Anderson et al., 2011; Cullen et al., 2011; Zhou and Nathans, 2014; Posokhova et al., 2015). In zebrafish, *gpr124* ICD as well as *gpr124* null alleles abrogate dorsal root ganglia (DRG) formation (Vanhollebeke et al., 2015; America et al., 2022). However, even full *Gpr124* knockout did not result in DRG severe neurogenesis defects in mouse (Fig. S4), suggesting differences in GPR124 function between mammals and zebrafish.

The sole angiogenic deficits in *Gpr124^ΔC/ΔC^* embryos were confined to the MGE, sparing the cortex, and manifested as glomeruloid malformations (Fig. 2D, middle column b) or reduced vascular density (Fig. 2D, middle column b′). Compared with severe and completely penetrant bilateral MGE glomeruloid malformations in *Gpr124^−/−^* embryos, the few MGE glomeruloid malformations in *Gpr124^ΔC/ΔC^* embryos were unilateral or absent (Fig. 2F). The MGE vascular density in *Gpr124^ΔC/ΔC^* was lower than in wild type but significantly higher than in *Gpr124^−/−^* (Fig. 2H). The expression of the BBB marker glucose transporter *Glut1* in brain endothelial cells was reduced in *Gpr124^ΔC/ΔC^* versus wild type, but had higher residual expression than in *Gpr124^−/−^* (Fig. 2I). Importantly, although BBB marker/WNT target gene expression was decreased in *Gpr124^ΔC/ΔC^*, this was not accompanied by defects in vascularization of the cortex (Fig. S5A,B). Overall, these *Gpr124^ΔC/ΔC^* phenotypes were clearly distinguishable from *Gpr124^−/−^*, suggesting that the GPR124 ICD is not required for developmental forebrain cortex angiogenesis, and that its deletion elicits a comparatively minor and variably penetrant MGE vascularization defect.

### GPR124 ICD deletion mutants retain endothelial WNT target gene expression

**Fig. 3.**
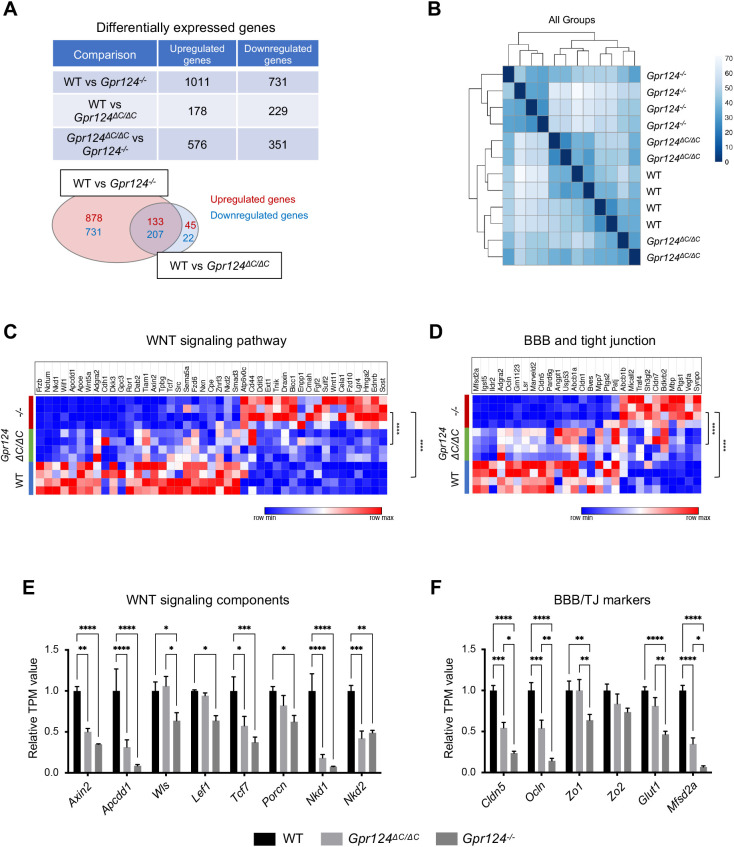
**GPR124 ICD deletion partially impairs WNT target gene expression in embryonic brain endothelial cells.** (A-F) Bulk RNA-seq analysis of FACS-purified CD31+ brain endothelial cells from wild-type, *Gpr124^ΔC/ΔC^* and *Gpr124^−/−^* E15.5 embryo forebrains in four biological replicates. (A) The number of differentially expressed genes based on absolute log_2_ fold change>1 and adjusted *P*-value <0.05. (B) Heatmap of sample-to-sample distance. The overall similarity among samples was assessed by the Euclidean distance between samples. The shorter the distance, the closer the relationship between samples. Samples were then clustered by using the imputed distance. (C,D) Heatmap of relative expression for (C) WNT signaling pathway (GO: 0016055) and (D) BBB/tight junction (GO: 0070160, 0060856 and 0035633) mRNAs that were differentially expressed between wild type and *Gpr124*^−/−^ (absolute log_2_ fold change>1, *P*<0.05). Two-sided *P*-values were calculated by two-way ANOVA, Tukey's multiple comparisons test. ****P<0.0001. (E,F) Relative TPM values of WNT signaling (E) and BBB/TJ components (F) from RNA-seq in wild type, *Gpr124^ΔC/ΔC^* and *Gpr124^−/−^.* Wild type, *n*=4; *Gpr124^ΔC/ΔC^*, *n*=4; *Gpr124*^−/−^, *n*=4. Data are mean±s.e.m. Two-sided *P*-values were calculated by two-way ANOVA, Tukey's multiple comparisons test. *P<0.05, **P<0.01, ***P<0.001 and ****P<0.0001.

To explore the impact of GPR124 ICD deletion on BBB transcriptional programs, we performed RNA-seq using brain endothelial cells isolated from wild-type, *Gpr124^ΔC/ΔC^* and *Gpr124^−/−^* E15.5 embryo forebrains in four biological replicates. The number of differentially expressed genes (upregulated, 178 genes; downregulated, 229 genes) in wild type versus *Gpr124^ΔC/ΔC^* was less than in wild type versus *Gpr124^−/−^* (upregulated, 1011 genes; downregulated, 731 genes) (Fig. 3A). Most of the genes that were differentially expressed in wild type versus *Gpr124^ΔC/ΔC^* overlapped with those in wild type versus *Gpr124^−/−^* (133 genes upregulated and 207 genes downregulated) (Fig. 3A). In a sample-to-sample distance heatmap, hierarchical clustering revealed that wild-type and *Gpr124^ΔC/ΔC^* were closely related in overall gene expression whereas *Gpr124^−/−^* formed a distinct cluster (Fig. 3B). For the differentially expressed genes between wild type, *Gpr124^ΔC/ΔC^* and *Gpr124^−/−^*, we focused on genes from the gene ontology (GO) terms ‘WNT signaling pathway’ (GO: 0016055) and some related to BBB or tight junctions (GO: 0070160, 0060856 and 0035633). *Gpr124^−/−^* knockout mice manifested strongly dysregulated WNT signaling and BBB/tight junction gene expression, which were not significantly altered in *Gpr124^ΔC/ΔC^* mice relative to wild type (Fig. 3C-F, Fig. S6A), consistent with minimal *Gpr124^ΔC/ΔC^* vascular phenotypes (Fig. 2). We also analyzed the WNT target mRNAs *Axin2* and *Apcdd1*, as well as the BBB-enriched mRNAs *Cldn5*, *Ocln*, *Zo1* (*Tjp1*), *Glut1* (*Slc2a1*) and *Mfsd2a* (Ben-Zvi et al., 2014; Chang et al., 2017; Cho et al., 2017; Boye et al., 2022). Expression of these genes was significantly downregulated by *Gpr124^−/−^* knockout, but milder reductions, if any, were typically observed in *Gpr124^ΔC/ΔC^* (Fig. 3E,F). The immunofluorescence staining revealed a decreased number of GLUT1-expressing CD31+ endothelial cells and impaired expression of GLUT1 and CLND5 in cortex and MGE (Fig. S5), consistent with RNA-seq showing hypomorphic partial impairment of WNT signaling (Fig. 3). Importantly, although BBB marker expression was decreased in *Gpr124^ΔC/ΔC^* cortex, this was not accompanied by any defects in vascularization, as the capillary networks in the cortex were normal (Fig. 2G). GO enrichment analysis of the differentially expressed genes revealed that *Gpr124^−/−^*, but not *Gpr124^ΔC/ΔC^*, mice exhibited dysregulation of gene expression relevant to cell migration, cell adhesion and angiogenesis (Fig. S7). We also analyzed adult brain endothelial cells from *Gpr124^flox/+^; Cdh5-CreER*, *Gpr124^flox/ΔC^; Cdh5-CreER* and *Gpr124^flox/−^; Cdh5-CreER* mice, that, upon *in vivo* tamoxifen treatment, yielded effective *Gpr124*^+/−^, *Gpr124^ΔC/−^* and *Gpr124*^−/−^ genotypes, respectively. Upon subsequent qRT-PCR analysis, alterations in WNT target mRNAs *Axin2*, *Apcdd1* and *Cldn5* between *Gpr124*^+/−^ and *Gpr124^ΔC^*^/−^ endothelium did not reach statistical significance, in contrast to more severe reductions in *Gpr124*^−/−^ (Fig. S6B). Thus, GPR124^ΔC^ protein is still competent to maintain endothelial WNT signaling and target gene expression.

### GPR124 regulates WNT signaling in a cell surface expression-dependent manner independent of the ICD

**Fig. 4.**
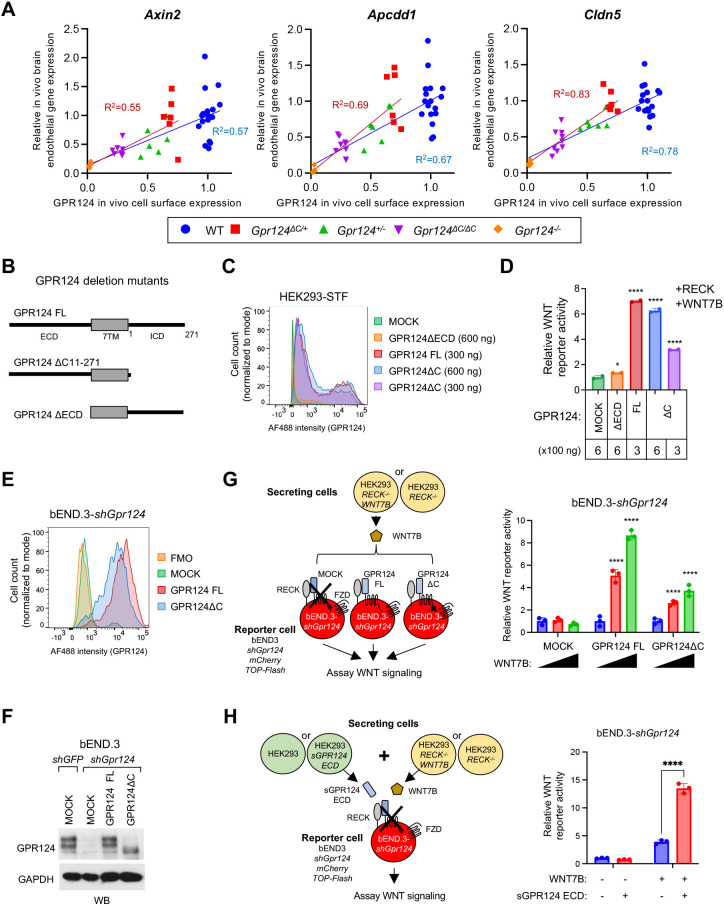
**GPR124 regulates WNT signaling in a cell surface expression-dependent manner that is independent of the GPR124 ICD.** (A) Correlation between cell surface GPR124 protein level and WNT target gene expression in embryonic brain endothelium. Plots were generated from cell surface expression (Fig. 1E) and gene expression assessed by RT-qPCR from E15.5 FACS-purified CD31+ brain EC (Fig. 3) from the indicated genotypes. Each value was normalized to wild-type control. Wild type, *n*=17 embryos; *Gpr124^ΔC/+^*, *n*=6; *Gpr124^+/−^*, *n*=6; *Gpr124^ΔC/ΔC^*, *n*=8; *Gpr124^−/−^*, *n*=5. (B) GPR124 FL, ICD deletion mutant and ECD deletion mutant transfection constructs. (C) FACS analysis of cell-surface GPR124 expression after transfection of the constructs in B. HEK293 STF cells were co-transfected with the indicated expression constructs (mock, 600 ng; *GPR124ΔECD*, 600 ng; *GPR124ΔC*, 300 or 600 ng; *GPR124 FL*, 300 ng per well) and *RECK* and *WNT7B*, and cultured for 2 days. (D) Relative WNT reporter activity in Super TOP-Flash (STF) canonical WNT/β-catenin reporter assay from C. (E) FACS analysis of cell-surface GPR124 expression in bEND.3-*shGpr124* cells after lentiviral overexpression of shRNA-resistant lenti-*GPR124 FL* and *GPR124ΔC*. (F) Immunoblotting analysis of E with anti-GPR124 (top panel) and anti-GAPDH (bottom panel). (G) Rescue of WNT7 signaling in GPR124-deficient bEND.3 reporter cells by GPR124 FL or GPR124ΔC secretor cells. bEND.3-*Gpr124 shRNA-TOP-Flash-mCherry* cells were co-cultured with increasing amounts of HEK293 *RECK^−/−^ WNT7B* or control HEK293 *RECK^−/−^* cells for 2 days (blue, no WNT7B cells; red and green represent HEK293 *RECK^−/−^ WNT7B*+bEND.3-*Gpr124 shRNA-TOP-Flash-mCherry* co-culture at 0.5:1 and 1:1 ratios, respectively). Firefly luciferase activity from TOP-Flash was normalized to the no WNT7B stimulation condition (right panel). (H) GPR124 ECD stimulates WNT7 signaling in GPR124-deficient bEND.3 cells. Here, bEND.3-*Gpr124 shRNA-TOP-Flash-mCherry* reporter cells were co-cultured with HEK293 *RECK^−/−^ WNT7B* or HEK293 *RECK^−/−^* control secretor cells, in addition to either secretor HEK293 cells expressing sGPR124 ECD or control HEK293. TOP-Flash luciferase activity was normalized to the no WNT7B, no sGPR124 ECD condition (right panel). Data are mean±s.e.m. of three independent experiments. Two-sided *P*-values were calculated by one-way ANOVA Dunnett's multiple comparisons test for D (versus mock), and two-sided *P*-values were calculated by two-way ANOVA, Dunnett's multiple comparisons test for G (versus left bar: no WNT7B stimulation in each) and H [versus control (blue bar)]. *P<0.05 and ****P<0.0001.

We next correlated differential cell surface expression levels of GPR124 WT and GPR124ΔC with WNT signaling activity in the corresponding mouse brain endothelium. The availability of multiple *Gpr124* mutants constituted an allelic series with progressively decreasing GPR124 cell surface expression as *Gpr124^+/+^* (wild type)>*Gpr124^ΔC/+^* > *Gpr124^+/−^* > *Gpr124^ΔC/ΔC^* > *Gpr124^−/−^* (Fig. 1E), which was then correlated to WNT target gene expression in FACS-sorted embryonic brain endothelium from corresponding E15.5 embryos (Fig. 3). In this allelic series, the mRNA expression of WNT target genes *Axin2* and *Apcdd1*, and the BBB marker *Cldn5* corresponded to the amount of cell-surface GPR124 expression, following the rank order *Gpr124^+/+^* (wild type)>*Gpr124^ΔC/+^*>*Gpr124^−/+^*>*Gpr124^ΔC/ΔC^*>*Gpr124^−/−^*, where both wild type and *Gpr124^ΔC/+^* attained maximal WNT signaling activity (Fig. 4A). The coefficient of determination R^2^, which describes the correlation between GPR124 cell surface expression versus WNT target gene expression in the allelic series of wild type, *Gpr124^+/−^*, *Gpr124^ΔC/ΔC^* and *Gpr124^−/−^*, was 0.57 for *Axin2*, 0.67 for *Apcdd1* and 0.78 for *Cldn5*. Similarly, R^2^ in the allelic series of *Gpr124^ΔC/+^*, *Gpr124^+/−^*, *Gpr124^ΔC/ΔC^* and *Gpr124^−/−^* was 0.55 for *Axin2*, 0.69 for *Apcdd1* and 0.83 for *Cldn5* (Fig. 4A). The R^2^ value of *Cldn5* indicated strong positive correlation with cell-surface GPR124 expression and the R^2^ value of *Axin2* and *Apcdd1* showed moderate positive association. These results suggest that the minor *Gpr124^ΔC/ΔC^* CNS vascular phenotypes are attributable to decreased cell surface GPR124 expression and do not obligately invoke impaired receptor signaling.

We also tested whether impaired GPR124^ΔC^ WNT signaling could correlate with decreased cell-surface expression in transient transfection assays. Specifically, the reduced cell surface expression of GPR124^ΔC^ relative to full-length GPR124 (GPR124 FL) was rescued by the transfection of increasing amount of GPR124^ΔC^ (600 ng) into HEK293 cells, in which the decreased GPR124^ΔC^ cell surface expression was normalized to the level of GPR124 wild-type protein (Fig. 4B-D). Under these equivalent cell surface expression conditions, canonical WNT reporter assays using TOP-Flash luciferase reporter in HEK293 cells revealed equivalent WNT7 signaling strength for both GPR124 FL and GPR124^ΔC^ in the presence of RECK (Fig. 4B-D), suggesting that GPR124 ICD is not essential for WNT7 signaling, paralleling results in primary mouse brain endothelium (Fig. 4A).

Furthermore, the activity of GPR124^ΔC^ versus GPR124 FL was compared upon expression in *Gpr124*-knockdown bEND.3 endothelial cells. We used previously described mouse brain endothelial cells with *Gpr124* knockdown targeting the 3′-UTR (Kuhnert et al., 2010), which were further lentivirally transduced with the TOP-Flash WNT reporter and mCherry. The resultant bEND.3-*Gpr124 shRNA-TOP-Flash-mCherry* cells comprised a *Gpr124-*knockdown background into which lentiviruses expressing shRNA-resistant human *GPR124 FL* and *GPR124ΔC* constructs lacking their 3′UTR were transduced (Fig. 4E,F). Graded WNT7 stimulation was achieved by co-culturing bEND.3-*Gpr124 shRNA-TOP-Flash-mCherry* cells with increasing amounts of HEK293 *RECK^−/−^ WNT7B* cells (Vallon et al., 2018), which maximize WNT7B secretion without interference from competitive WNT7B binding to RECK. Both *GPR124 FL* and *GPR124^ΔC^* upregulated canonical WNT reporter activity in FACS-purified bEND.3-*Gpr124 shRNA-TOP-Flash-mCherry* cells in a WNT7 dose-dependent manner, affirming the signaling competence of GPR124^ΔC^ (Fig. 4G). We were technically unable to titrate lentiviral infections to achieve equivalent cell surface expression of GPR124 FL and GPR124^ΔC^. Thus, although both GPR124 FL and GPR124^ΔC^ exhibited signaling competence, the expected impaired expression of GPR124^ΔC^ versus GPR124 FL corresponded to decreased GPR124^ΔC^ WNT reporter activity (Fig. 4G).

Given the lack of effects of ICD deletion, and previous studies indicating that the GPR124 ECD promotes signaling in HEK293 cells (Vallon et al., 2018; America et al., 2022), we assessed whether the GPR124 ECD was sufficient to mediate WNT7 signaling in a more physiological endothelial cell context. Thus, we examined whether the GPR124 ECD could rescue the absence of endogenous GPR124 in *Gpr124*-knockdown mouse brain endothelial bEND.3 cells. Accordingly, bEND.3-*Gpr124 shRNA-TOP-Flash-mCherry* cells were simultaneously co-cultured with both HEK293 cells overexpressing the soluble GPR124 ECD (sGPR124 ECD) and HEK293 *RECK^−/−^* WNT7B cells. Notably, sGPR124 ECD was indeed sufficient to induce the TOP-Flash reporter in a WNT7B-dependent manner in *Gpr124*-knockdown bEND.3 cells lacking endogenous GPR124 (Fig. 4H). Together, the ability of the GPR124 ECD (Fig. 4H) and GPR124^ΔC^ (Fig. 4G) to rescue WNT7 signaling in *Gpr124*-knockdown bEND.3 cells, combined with the lack of *in vivo* BBB and CNS angiogenesis phenotypes in *Gpr124^ΔC/ΔC^* embryos, neonates and adults (Figs 1-3) suggests a mechanism of mammalian GPR124 action that primarily uses the ECD rather than the ICD.

## DISCUSSION

The BBB stringently regulates the passage of nutrients and macromolecules into the CNS, and BBB dysfunction underlies diverse pathologies, including stroke, neoplasia and neurodegeneration (reviewed by Rada et al., 2023; Wu et al., 2023). Despite the well-established GPR124/RECK/WNT7 essential regulation of developmental and pathological brain angiogenesis and BBB function, the molecular mechanism of GPR124 action remains poorly understood (Stenman et al., 2008; Daneman et al., 2009; Cho et al., 2017; Eubelen et al., 2018; Vallon et al., 2018). Discrepancies in GPR124 ICD function between mammals and zebrafish have been inferred from *in vitro* cell culture and zebrafish CNS angiogenesis models. During zebrafish embryogenesis, *gpr124* ICD mutants cannot rescue *gpr124-*null brain angiogenesis phenotypes (Eubelen et al., 2018; America et al., 2022). Specifically, the zebrafish GPR124 ICD has been reported to bind DVL, which then associates with FZDs, whereas mammalian GPR124 ICD does not bind to DVL (Eubelen et al., 2018; America et al., 2022). The C-terminal PDZ domain binding motifs (E-T-X-V) of both zebrafish and mammalian GPR124/FZD have been suggested as binding sites for the MAGUK adaptor proteins DLG1, DLG4 and MAGI3, as an additional GPR124-FZD bridging mechanism (Posokhova et al., 2015; America et al., 2022). However, using *in vitro* transfection assays, mammalian GPR124 ICD deletion mutants are still competent to enhance WNT7 signaling, in contrast to analogous zebrafish mutants (Posokhova et al., 2015; Vallon et al., 2018; America et al., 2022). We thus deleted the GPR124 ICD in mice to definitively address the function of this domain and determine its *in vivo* relevance to mammalian CNS angiogenesis.

Murine GPR124 null alleles are well established to elicit a pronounced embryonic lethal CNS-specific phenotype of impaired forebrain angiogenesis, hemorrhagic glomeruloid malformations, BBB defects and endothelial *Glut1* loss (Kuhnert et al., 2010; Anderson et al., 2011; Zhou and Nathans, 2014; Posokhova et al., 2015; Chang et al., 2017). However, GPR124 ICD deletion in *Gpr124^ΔC/ΔC^* mice did not induce embryonic lethality and live pups could be born. Some *Gpr124^ΔC/ΔC^* mice even survived to adulthood with lifespan comparable to littermate controls, although with slight reductions in body weight. Approximately 80% of *Gpr124^ΔC/ΔC^* mice died postnatally with cyanosis and respiratory compromise, but crucially possessed intact BBB integrity, as opposed to *Gpr124^−/−^* and *Reck^−/−^* mice (Anderson et al., 2011; Cho et al., 2017). One of many contributors to BBB-independent postnatal death in *Gpr124^ΔC/ΔC^* pups could be cleft palate, as reported in *Gpr124*-null mice (Anderson et al., 2011). Approximately 40% of *Gpr124^ΔC/ΔC^* P0 pups indeed exhibited cleft palate, and aspirated milk was present in the lungs of 12.5% of *Gpr124^ΔC/ΔC^* pups, which could underlie respiratory compromise. Potentially, altered MGE development from neurogenesis secondary to impaired angiogenesis and tissue damage could also contribute to subsequent postnatal death.

Strikingly, *Gpr124^ΔC/ΔC^* embryos exhibited normal cerebral cortex CNS angiogenesis without hemorrhage, in marked contrast to forebrain avascularity and glomeruloid malformations in *Gpr124^−/−^* null mice. Furthermore, *Gpr124^ΔC/ΔC^* neonates did not show BBB leakage, suggesting that, although WNT signaling is slightly decreased in *Gpr124^ΔC/ΔC^* mice, residual WNT signaling activity still exceeds a threshold required for CNS angiogenesis and BBB function (Fig. S8), consistent with our previous proposal of a WNT signaling threshold model for adult BBB function (Chang et al., 2017). The only observed vascular phenotype in *Gpr124^ΔC/ΔC^* embryos was a mild and variably penetrant CNS angiogenesis defect restricted to the MGE. However, we cannot unequivocally attribute the MGE phenotype to ICD deletion because the GPR124^ΔC^ mutant protein was expressed at less than wild-type levels. Indeed, as the MGE is the most sensitive brain region to GPR124/RECK signaling alteration (Zhou and Nathans, 2014; Cho et al., 2017), overall reduction in GPR124 protein levels, as opposed to ICD deletion, could conceivably underlie the *Gpr124^ΔC^* phenotype. *In vivo*, the degree of cell surface GPR124 expression in the *Gpr124^-^, Gpr124^ΔC^* and *Gpr124^+^* allelic series exhibited a direct correlation with brain endothelial expression of WNT target and BBB mRNAs. These data contrast with a recent study in which mammalian (mouse and human) GPR124 ICD deletion mutants failed to completely restore the CNS angiogenesis phenotype of *gpr124* knockdown zebrafish (∼60% rescue efficiency) and cell surface expression of the deletion constructs *in vivo* was not quantified (America et al., 2022). Conceivably, these differences could be attributable to species-specific signaling when mammalian GPR124 is expressed in zebrafish versus a more physiologically relevant mammalian context.

Overall, GPR124 ICD deletion mice still maintained sufficient endothelial WNT signaling to undergo normal cerebral cortex angiogenesis and did not phenocopy well-established *Gpr124* KO severe CNS vascular defects (Kuhnert et al., 2010; Anderson et al., 2011; Cullen et al., 2011; Zhou and Nathans, 2014), despite reduced Gpr124^ΔC^ protein expression (Fig. S8B). In mice, the ICD could confer stability of the GPR124 protein instead of mediating intrinsic signaling functions; however, this by no means excludes essential ICD functions in zebrafish or non-essential activities in mammals. In contrast to the ICD, an essential effector activity of the GPR124 ECD is consistently supported by chimeric zebrafish/mouse GPR124 protein studies and signaling activation by recombinant GPR124 ECD (Vallon et al., 2018; America et al., 2022). The present data support this model, as GPR124 ECD indeed potentiated WNT7 signaling in *Gpr124*-knockdown bEND.3 cells, although mechanisms of GPR124 ECD-mediated WNT7 signaling remain unclear and further analyses will be required to determine functional interactions with RECK and FZD/LRP. As GPR124 is a member of the adhesion G-protein-coupled receptor (aGPCR) family, which exhibits functional cooperativity between the ECD, 7TMD and ICD motifs (Vizurraga et al., 2020; Lala and Hall, 2022), our results provide precedent for signaling mediated at least partially through extracellular domains. Further investigations into this ECD signaling mechanism could yield an increased understanding of adhesion G-protein-coupled receptor function, as well as identify potentially druggable interfaces for therapeutic targeting.

## MATERIALS AND METHODS

### Generation of the Gpr124^ΔC^ allele

Gpr124-V5-stop ΔC mice were generated by CRISPR/Cas9-induced homologous recombination in single cell zygotes. Guide-RNA sequences used were as follows: 5′-AGCCCGGACATCTCTACGTC-3′ and 5′-AGCAGGCGCGCCAGGAAGCC-3′. A two-hundred nucleotide single-stranded oligo donor template was designed to insert the V5-STOP sequence in frame with the last transmembrane domain encoded by exon 19 and also introduced silent PAM site mutations to prevent further editing of the recombined allele. The sequence of this donor template is as follows: 5′-TGTCTGTACGGCGTGGCAGCTTCAGCTCTTGGTCTGTTTGTCTTCACTCACCACTGTGCTAGACGTAGAGATGTTCGGGGTAAGCCTATCCCTAACCCTCTCCTCGGTCTCGATTCTACGTAAGCTTCCTGGCGCGCCTGCTGCCCTCCTGCTTCGCCCTCGGCCTCCCATGTCCCAGCCCGGGCCCTGCCGACTGCTAC-3′. gRNA, tracrRNA, the single stranded oligo donor template, and Cas9 protein were purchased from Integrated Data Technologies, and the gRNA-tracrRNA-Cas9 complex was formed according to manufacturer instructions. Super-ovulated C57Bl/6 females underwent timed matings and zygotes were harvested at the single cell stage. The CRISPR/Cas9 complex+donor template was microinjected into the zygote cytoplasm and cultured overnight in KSOM medium at 37°C. Viable two-cell embryos were transferred to the oviducts of pseudo-pregnant recipient dams. Microinjections and transgenesis were performed at the Penn Vet Transgenic Mouse Core. Subsequently, mating of F0 founder animals to B6DF1 females generated germline F1 animals, which were subsequently sequenced to confirm the desired mutation and establish the colony. The presence of the V5 tag was confirmed by genomic PCR using the following primers: 5′- TGTGTAGCTGTCTGTACGGC-3′ (forward) and 5′-CCATCCTCTGTAGCAGTCGG-3′ (reverse) with the expected products of 217 bp (*Gpr124^ΔC^* allele) and 172 bp (wild-type allele).

### Mice

Heterozygous *Gpr124^ΔC/+^* and *Gpr124^lacZ/+^* (referred to as *Gpr124^+/−^*) (Kuhnert et al., 2010) parents were intercrossed to generate homozygous *Gpr124^ΔC/ΔC^* and *Gpr124^−/−^* mice, respectively. E13.5 or E15.5 mouse embryos were obtained by setting up breeding pairs for timed pregnancies. The morning a female was found with a vaginal plug was considered E0.5. For adult mouse experiments, *Gpr124^ΔC/+^* and *Gpr124^+/−^* mice were crossed with mice bearing *Gpr124^flox^* allele (Chang et al., 2017) and the endothelial specific tamoxifen-inducible driver *Cdh5-CreER* (Wang et al., 2010). *Gpr124^flox/−^; Cdh5-CreER* and *Gpr124^flox/ΔC^; Cdh5-CreER* mice were administered with four doses of tamoxifen (2 mg/10 g body weight) (Sigma-Aldrich) in corn oil via oral gavage every other day for 7 days. Mice were allowed to recover from tamoxifen treatment for 3-4 weeks before experiments. Mice were housed in 12 h light and dark cycles in a pathogen-free animal facility. All animal experiments were performed in accordance with procedures approved by the Institutional Animal Care and Use Committee (IACUC) at Stanford University.

### Cell lines and maintenance

HEK293 (ATCC #CRL-1573), bEND.3 subline cells generated as described below were maintained in DMEM supplemented with 10% FBS and 100 μg/ml Normocin (InvivoGen) at 37°C and 5% CO_2_. Cells were tested for mycoplasma using PCR (25235, LiliF) and found to be negative.

### Primary brain endothelial cell culture

Adult mouse brains from wild-type, *Gpr124^ΔC/ΔC^* and *Gpr124^flox/flox^*; *Cdh5-CreER* mice were minced and digested with type IV collagenase (400 U/ml, Worthington), dispase (1.2 U/ml, Worthington) and DNase I (32 U/ml, Worthington) at 37°C for 30-40 min with pipetting every 10 min until adequate disaggregation, as previously described (Chang et al., 2017). FBS was added to the disaggregation reaction and the disaggregated cells were filtered through a 70 μm cell strainer and washed with 1× ice-cold PBS. After centrifugation at 400 ***g*** for 5 min, cell pellets were resuspended with 20% BSA and centrifuged at 1000 ***g*** at 4°C for 25 min to remove the myelin. Cells from one brain seeded into two wells of a six-well plate or 60 wells of a 96-well plate after coating the plates with 10 μg/ml fibronectin, and cultured in EGM-2-MV medium (CC3202, Clontech) until confluent (∼7 days). Cell culture medium was supplemented with 4 μg/ml puromycin and 1 μM 4-hydroxytamoxifen (6278, Sigma-Aldrich) for the first 3 days to kill all the other cell types, except brain ECs, and to induce endothelial-specific *Gpr124* knockout in *Gpr124^flox/flox^*; *Cdh5-CreER* cells. Wild-type and *Gpr124^ΔC/ΔC^* cells isolated in parallel received the same treatment. Medium was replaced every 2-3 days. After 7 days in culture, cells were analyzed for cell surface ELISA or lysed for immunoblotting.

### Tissue processing and immunofluorescence staining

E13.5 embryos and postnatal day 0 (P0) brains were harvested and fixed with 4% PFA in PBS at 4°C overnight and then transferred to 30% sucrose in PBS at 4°C overnight before being embedded in OCT and frozen on dry ice. Frozen tissues were sliced at 60 μm in free-floating sections using a cryostat and collected into a multi-well plate containing PBS. Sections were rinsed three times with PBS for 5 min each and incubated in blocking buffer (5% goat serum+0.3% Triton X-100 in PBS) at room temperature for 60 min. Sections were incubated with the following primary antibodies: hamster anti-CD31 (1:100, MAB1398Z, Millipore), rabbit anti-GLUT1 (1:200, RB-9052-P1, Thermo Fisher Scientific), rabbit anti-CLDN5 (1:100, 341600, Zymed), rabbit-anti-ZO1 (1:100, 617300, Zymed), rabbit anti-NeuN (1:100, ZRB377-4, Millipore) and rabbit-anti Islet1 (1:100, ab20670, Abcam) diluted in antibody dilution buffer (1% BSA+0.3% Triton X-100 in PBS) on a shaker at 4°C overnight. Sections were washed more than six times with PBS-T (0.1% Triton X-100 in PBS) over the course of 6 h, and subsequently incubated with the following secondary antibodies: FITC-labeled goat anti-rabbit IgG (1:500, 111-545-144, Jackson ImmunoResearch), Cy3-labeled goat anti-hamster IgG (1:500, 127-165-160, Jackson ImmunoResearch), FITC-labeled goat anti-rabbit IgG (1:500, 111-545-144, Jackson ImmunoResearch) and DAPI on a shaker at 4°C overnight in the dark. The next day, sections were washed more than six times with PBS-T over the course of 6 h and mounted on to slides using Vectamount AQ Aqueous Mounting Medium (H5501, Vector Laboratories). Sections were imaged using a LSM880 or LSM900 confocal microscope and processed with ImageJ. For quantification of relative vascular density, 60 μm coronal sections of embryonic brains were stained with anti-CD31 and anti-GLUT1. Confocal images were scanned at 10 μm intervals along the *z*-axis, on which two images were *z*-stacked. The following areas of the *z*-stacked images were analyzed: cortex (450×600 μm) and MGE (450×600 μm). All the blood vessels within the designated volume were traced using Adobe Illustrator software. The lengths of the traced vessels were quantified by calculating pixel coverage as a fraction of the total area using ImageJ, as previously described (Cho et al., 2017).

### Sulfo-NHS-Biotin and sodium fluorescein administration

Pups were injected intraperitoneally with 100 μl of EZ-link-sulfo-NHS-biotin (20 mg/ml, 21217, Thermo Fisher Scientific) in PBS 30 min before sacrifice. Brains were fixed with 4% PFA in PBS at 4°C overnight and then transferred to 30% sucrose in PBS at 4°C overnight before being embedded in OCT. Frozen sections (60 μm) were stained with Cy5-strepavidin (1:500, 016-170-084, Jackson ImmunoResearch) and imaged using a LSM880 confocal microscope. Pups were injected intraperitoneally with 100 μl of sodium fluorescein, NaFl (10 mg/ml, F6377, Signa-Aldrich) in PBS and animals were sacrificed 30 min later. Frozen sections were made as described above and imaged after mounting with DAPI by Keyence microscope (BZ-X710).

### Histological analysis

Live and dead newborn mice were collected at P0 (live pups were euthanized by CO_2_). Pups were fixed with 10% neutral buffered formalin at room temperature, transferred to 70% ethanol, routinely processed, embedded in paraffin and sectioned at 5 μm. Tissue sections were mounted on glass slides and routinely stained with Hematoxylin and Eosin (H&E). Stained tissue sections were visualized with an Olympus BX43 bright-field microscope. Photomicrographs were captured using an Olympus DP27 digital camera and the Olympus cellSens software.

### FACS analysis and isolation of endothelial cells

E15.5 forebrains were digested with Liberase I (25 μg/ml, Roche) and DNase I (10 U/ml) in PBS containing Ca^2+^ and Mg^2+^ for 30 min with pipetting every 10 min. Adult mouse brains were minced and disaggregated as described above. The cells were then resuspended in 3% FBS in PBS. Cells were stained using APC-rat anti-mouse CD31 (1:100, 17-0311-82, eBiosciences), PE-rat anti-mouse CD140b (PDGFRB) (1:100, 12-1402-81, eBiosciences), PECy7 rat anti-mouse CD45 (1:100, 552848, BD Pharmingen), rabbit anti-GPR124 ECD (1:100; Vallon et al., 2018) antibody with secondary antibody AF488-goat anti-Rabbit IgG (H+L) (111-545-144, Jackson ImmunoResearch) and 7-AAD (Invitrogen). Endothelial cell fractions (CD31+/CD140b−/CD45−/7AAD−) were sorted by a BD FACSAria II (BD Biosciences) for RNA analysis. FACS data were analyzed using FlowJo software (BD Life Sciences). Cell surface GPR124 ratio was calculated as (sample GPR124 MFI minus GPR124 FMO MFI)/(wild-type GPR124 MFI minus GPR124 FMO MFI).

### Real-time quantitative PCR (RT-qPCR)

Total RNA was isolated from cells using PicoPure RNA Isolation Kit (Applied Biosystems) and cDNA was synthesized using iScript Reverse Transcription Supermix (Bio-Rad). RT-qPCR was performed with Power SYBR Green assay (Applied Biosystems) after pre-amplification (SsoAdvanced PreAmp Supermix, Bio-Rad). Relative RNA expression was calculated using a standard curve method and normalized by *Gapdh* or *Actb*.

### RNA-seq

Total RNA was isolated from sorted CD31+ embryonic brain endothelial cells using the Arcturus PicoPure RNA Isolation Kit. Library preparation with poly(A) selection, 150 bp paired end sequencing on an Illumina HiSeq 2500 and bioinformatic analysis were performed by Genewiz. Transcript abundances are presented as transcripts per million (TPM). Differentially expressed genes were identified based on absolute log_2_ fold change>1 and an adjusted *P*-value<0.05. Heatmaps were generated using Morpheus (https://software.broadinstitute.org/morpheus). The data have been deposited in GEO under accession number GSE225367.

### Immunoblot staining

Tissue lysate preparation and immunoblot analyses were performed using standard methods. Briefly, cells were harvested in RIPA buffer (TBS, 1% Triton X-100, 0.5% sodium deoxycholate and 0.1% SDS) or lysis buffer [50 mM Tris-HCl (pH 6.8), 1% SDS, 1 mM EDTA] containing protease inhibitor cocktail (cOmplete Mini, Roche) and centrifuged at 5000 ***g*** for 10 min to remove debris. Protein concentration was assessed using BCA Kit (Bio-Rad). Samples were supplemented with NuPage LDS sample buffer (Thermo Fisher Scientific) containing 5% 2-mercaptomethanol. NuPage 4-12% Bis-Tris Gels (Thermo Fisher Scientific) were used for SDS-PAGE. PageRuler Plus Prestained Protein Ladder (Thermo Fisher Scientific) was used as a molecular weight marker. Separated proteins were transferred to PVDF membranes (EMD Millipore) and membranes were blocked with 5% non-fat dry milk in TBS/0.05% Triton-X (TBST/milk). Membranes were incubated overnight with the following primary antibodies: rabbit anti-GPR124 (1:2000; Vallon et al., 2018) and rabbit anti-GAPDH (1:2000, 5174S, Cell Signaling Technology) diluted in TBST/milk. After antibody incubations, membranes were washed three times with TBST for 10 min each and incubated with the secondary antibody HRP-conjugated donkey anti-rabbit IgG (H+L) (1:5000, 711-035-152, Jackson ImmunoResearch). Bound antibodies were visualized using SuperSignal West Pico/Femto Chemoluminescent Substrates (Thermo Fisher Scientific) and exposure of autoradiography and chemiluminescence films for western blots (MidSci).

### GPR124 FL and Gpr124ΔC11-271 overexpression in Gpr124-knockdown bEND.3 cells

Murine *Gpr124* knockdown-bEND.3 cells were previously generated by infection with shRNA containing lentivirus targeting *Gpr124* (SHCLNV-NM_05404, Sigma-Aldrich) (Kuhnert et al., 2010). The cells were infected with lentivirus 7TFC coding 7xTcf-Firefly luciferase with SV40-mCherry. 7TFC was a gift from Roel Nusse (Stanford University, CA, USA) (Addgene plasmid # 24307) (Fuerer and Nusse, 2010). For GPR124 overexpression, *Gpr124* FL (Vallon and Essler, 2006) and *Gpr124*ΔC11-271 (Vallon et al., 2018) were cloned into a lentivirus vector (CD515B-1, System Biosciences). The bEND.3- *shGpr124*-7TFC (*TOP-Flash-mCherry*) cells were infected with lentivirus GPR124 or GPR124ΔC11-271 and selected with hygromycin.

### Canonical WNT reporter assay

HEK293 STF cells (ATCC, CRL-3249) were plated on six-well plates. The following day, fresh culture medium was replaced, and cells were transfected with expression plasmids (300-600 ng of DNA per well) using the calcium phosphate precipitation method. The DNA master mix included: 15 ng of the internal control Renilla luciferase plasmid (pRL-TK, Promega), 600 ng of p3xFLAG-CMV-9-SNAPf-RECK, pcDNA-WNT7B and 300 ng or 600 ng of p3xFLAG-CMV-9-GPR124 FL, Gpr124ΔC11-271 (Vallon et al., 2018). Control vector was used to normalize all transfections to 600 ng. For sGPR124 experiments, HEK293 *RECK^−/−^* cells were transfected with RECK, WNT7B, STF reporter and pRL-TK, as previously described (Vallon et al., 2018). 48 h post-transfection, cells were lysed with 1× Passive Lysis buffer (Promega) and luciferase activities (Firefly and Renilla) were measured using the Dual-Luciferase Reporter Assay System (Promega). Firefly luciferase (STF) activity was normalized with Renilla luciferase activity and is shown relative to the activity in control. For brain endothelial cell canonical WNT reporter assay, GPR124 FL and GPR124ΔC11-271 overexpressing *Gpr124*-knockdown bEND.3-7TFC cells were plated at 4.75×10^4^ cells in 24-well plate and co-cultured with WNT7B stably expressing cells (HEK293 *RECK^−/−^ WNT7B*) (Vallon et al., 2018) at 0, 1.185, 2.37 and 4.75×10^4^ cells for 2 days. Control cells (HEK293 *RECK^−/−^* cells) were supplemented to a total of 4.75×10^4^ cells. For sGPR124 ECD experiments, bEND.3-*shGpr124*-7TFC (*TOP-Flash-mCherry*) cells were co-cultured with WNT7B-expressing cells (HEK293 *RECK^−/−^ WNT7B*) and sGPR124 ECD-expressing cells (HEK293-Flag-*sGPR124* ECD) (Vallon et al., 2018). Cells were trypsinized and resuspended with 3% FBS in PBS. 10,000 mCherry+ cells were sorted by BD FACSAria II and analyzed with the Luciferase Assay System (Promega).

### Cell-based ELISA

Confluent primary cultured mouse brain endothelial cells cultured in 96-well plates were fixed with 4% formaldehyde in PBS for 15 min, washed three times with PBS for 5 min each, and blocked with 5% normal donkey serum in PBS for 1 h. For permeabilization, blocking buffer had been supplemented with 0.3% Triton X-100. Cells were incubated with rabbit anti-mouse GPR124 ECD antibody or mouse anti-β-actin antibody (A1978, Sigma-Aldrich) at 1 μg/ml in PBS/1% BSA for 1 h at room temperature or overnight at 4°C. Cells were washed as before and incubated with HRP-conjugated donkey anti-rabbit or mouse IgG antibody diluted 1:10000 in PBS/1% BSA for 1 h. Cells were washed as before and 1-Step Ultra TMB Substrate (Thermo Fisher Scientific) was added. The reaction was stopped with 1 M H_2_SO_4_ and OD450 was measured using a plate reader. Background signal (cells only incubated with secondary antibody) was subtracted from specific signal and GPR124 signal was normalized to β-actin signal.

### Statistical analysis

Statistical analysis was performed using GraphPad Prism 9 (www.graphpad.com). Results were expressed as the mean±s.e.m. (standard error of mean). Statistical significance was determined by two-tailed *P*-values calculated by the indicated statistical analysis methods in the figure legends, including chi-square test, Tukey's multiple comparisons test and Dunnett's multiple comparisons test. Significance from these tests is represented by *P<0.05, **P<0.01, ***P<0.001, and ****P<0.0001.

## Supplementary Material

10.1242/develop.202794_sup1 Supplementary information
