# Supplementary material for: GPR124 regulates murine brain embryonic angiogenesis and BBB formation by an intracellular domain-independent mechanism
Source: Development. 2024 Jun 17;151(11):dev202794. doi: 10.1242/dev.202794 (PMC11213517; doi:10.1242/dev.202794)
Supplement: Supplementary information [file develop-151-202794-s1.pdf]

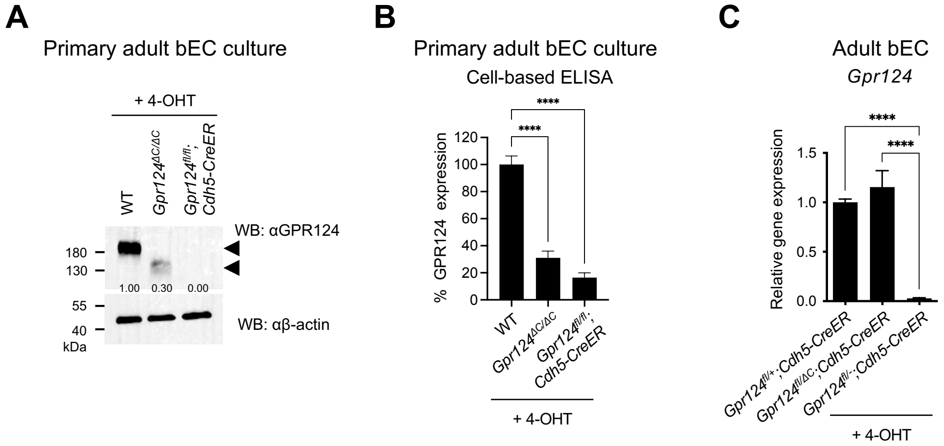

Fig. S1. GPR124 expression in adult brain endothelial cells.

(A-B) GPR124 expression in primary adult brain endothelial cells from the indicated genotypes, as assessed by immunoblotting with GPR124 densitometry normalized to β-actin (A) and cell-based ELISA as normalized by control WT (B). Data represent mean ± SEM of 3 technical replicates. (C) *Gpr124* expression, as assessed by RT-qPCR, in sorted brain endothelial cells from adult *Gpr124$^{fl/+}$; Cdh5-CreER, Gpr124$^{fl/\Delta C}$; Cdh5-CreER* and *Gpr124$^{fl/-}$; Cdh5-CreER* mouse forebrains. Tamoxifen was administered into mice 4 doses every other day for 1 week 3-4 weeks before experiments. Each value was normalized by *Gapdh* and littermate control. *Gpr124$^{fl/+}$; Cdh5-CreER*; n=7, *Gpr124$^{fl/\Delta C}$; Cdh5-CreER* ; n=5, *Gpr124$^{fl/-}$; Cdh5-CreER*; n=5. Data represent mean ± SEM. Two-sided P-values were calculated by one way ANOVA Tukey's multiple comparisons test. ****p<0.0001.

Development • Supplementary information

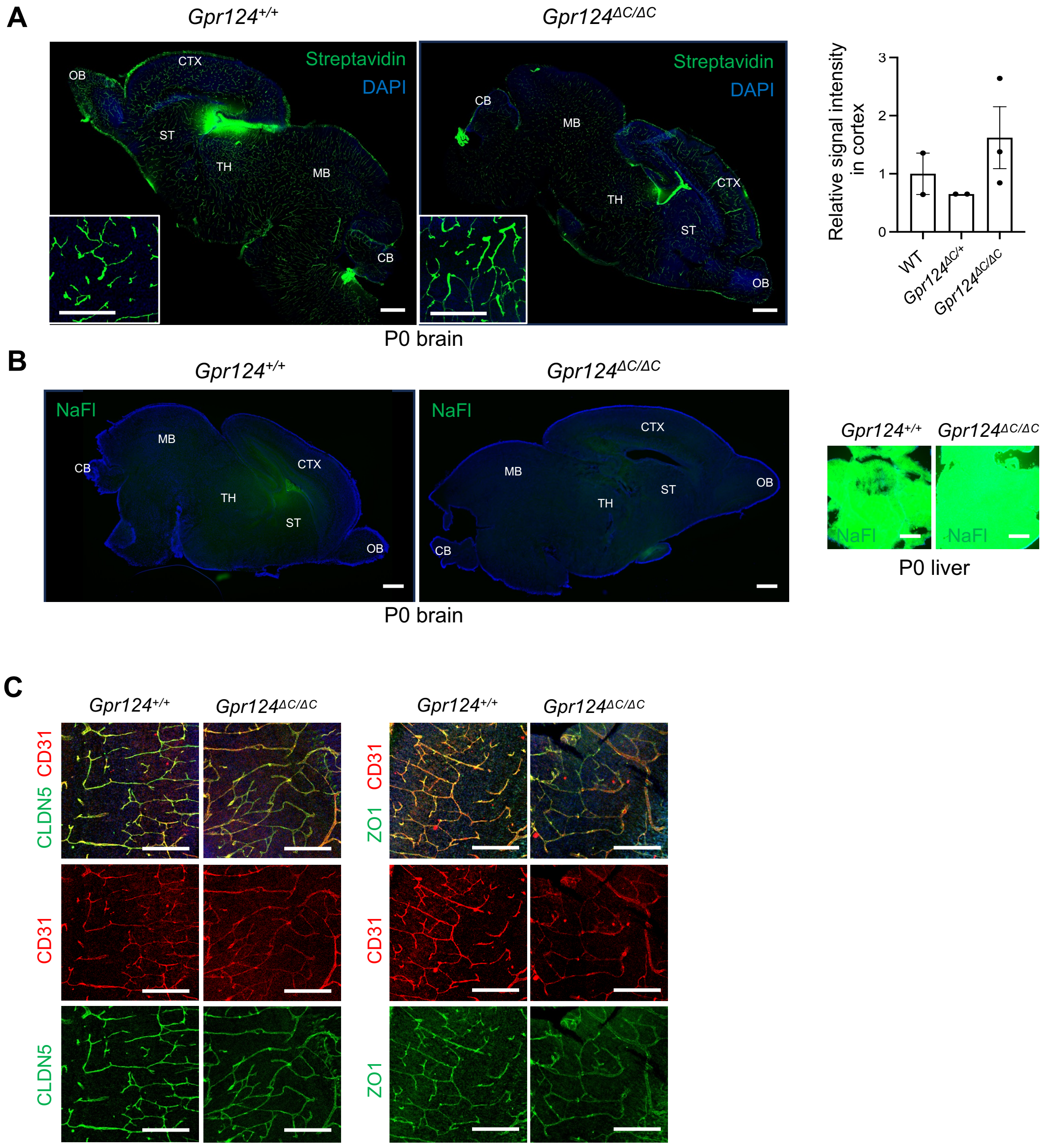

Development • Supplementary information

Fig. S2. BBB integrity in GPR124 ICD deletion pups. (A) Sagittal sections of sulfo-NHS-biotin-injected P0 mouse brains from the indicated genotypes. Pups were injected intraperitoneally with 100 µl of 20 mg/ml EZ-link-sulfo-NHS-biotin in PBS and harvested after 30 minutes. Frozen sections were stained with Cy5-strepavidin to detect biotin. Scale bar, 500 µm. Cortex regions are shown at higher magnification in the box. Scale bar, 200 µm. Fluorescent signal in cortex (100 x 100 µm) was quantified by ImageJ (right panel). WT; n=2, *Gpr124*$^{\Delta C/+}$; n=2, *Gpr124*$^{\Delta C/\Delta C}$; n=3. (B) Sodium fluorescein (NaFl) leakage in P0 pup brains and livers. Pups were injected i.p. with 100 µl of NaFl in PBS and harvested after 30 minutes. Sections were imaged after mounting with DAPI. Liver sections were also imaged in the same setting as a leakage positive control. Left panels, brain sections. Right panels, liver sections. Scale bar, 500 µm. CTX: cerebral cortex, CB: cerebellum, MB: midbrain, ST: striatum, TH: thalamus, OB: olfactory bulb. (C) IF staining of CD31 and BBB markers in cortex of P0 pups. Tight junction proteins, CLDN5 and ZO1. Scale bar, 200 µm.

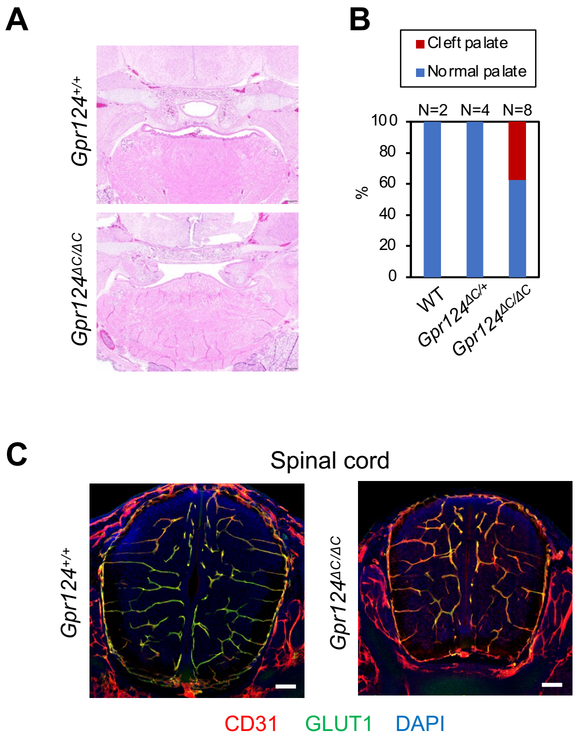

Fig. S3. Postnatal and embryonic GPR124 ICD deletion phenotypes. (A) H&E-stained coronal sections of P0 pups of the palatal region revealing incomplete fusion in *Gpr124*$^{\Delta C/\Delta C}$ vs. WT. Scale bar, 200 μm. (B) Frequency of cleft palate phenotype in the indicated number of pups stratified by genotypes. (C) IF staining of spinal cord sections in E13.5 embryos. Scale bar, 100 μm.

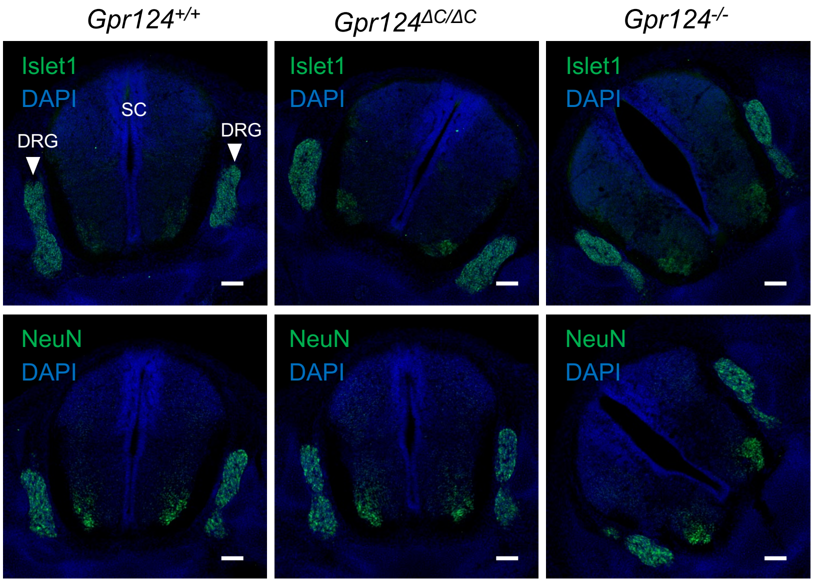

Fig. S4. Neuronal marker expression in E13.5 DRG. Immunofluorescence staining of Islet1 and NeuN in spinal cord sections in E13.5 embryos of the indicated genotypes. Scale bar, 100 µm. SC: spinal cord, DRG: dorsal root ganglia.

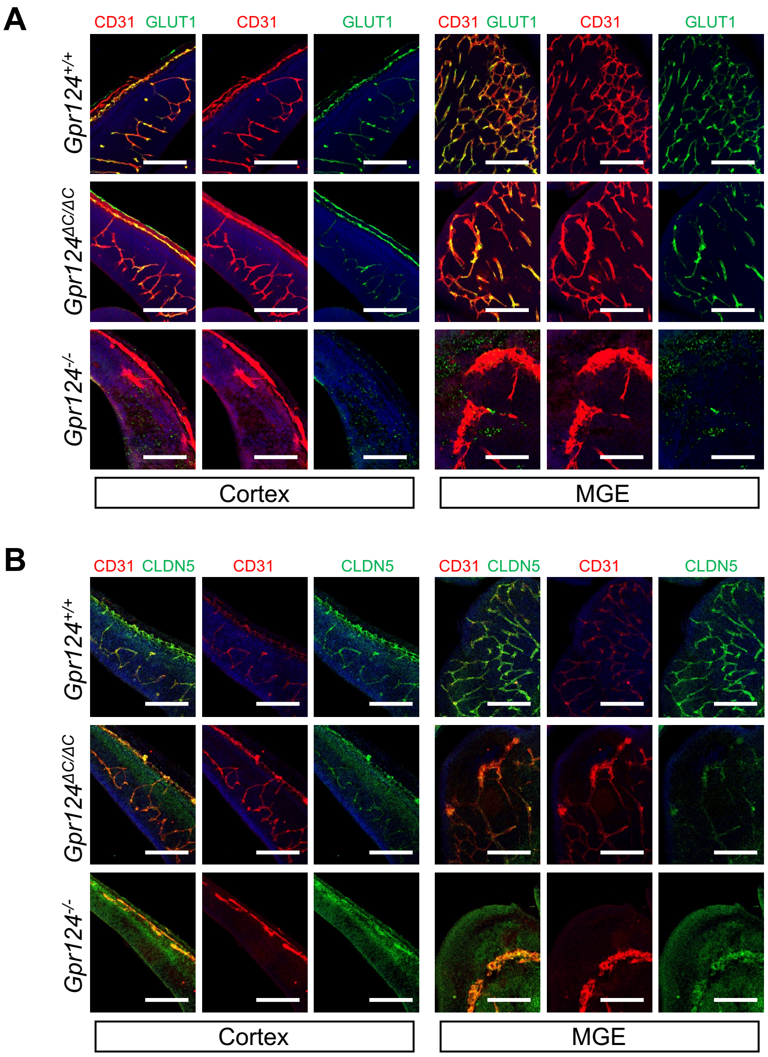

Fig. S5. BBB marker expression in E13.5 brain endothelial cells.
Immunofluorescence staining of brain coronal sections in E13.5 embryos of the indicated genotypes. (A) CD31, GLUT1, DAPI. Single–labeled images from Fig. 2D. (B) CD31, CLDN5, DAPI. Scale bar, 200 μm.

Development • Supplementary information

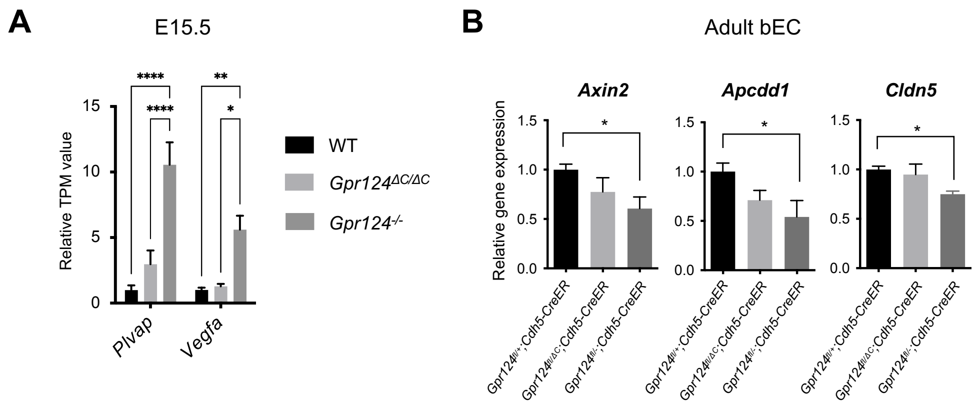

Fig. S6. WNT target gene expression in *Gpr124*$^{\Delta C/\Delta C}$ embryos and adult brain endothelial cells.(A) Relative TPM (transcripts per million) values of *Plvap* and *Vegfa* from RNA-seq in WT, *Gpr124*$^{\Delta C/\Delta C}$ and *Gpr124*$^{-/-}$ E15.5 embryos from Fig. 3. WT; *n*=4, *Gpr124*$^{\Delta C/\Delta C}$; *n*=4, *Gpr124*$^{-/-}$; *n*=4. (B) Expression of *Axin2, Apcdd1, Cldn5* and *Gpr124*, as assessed by RT-qPCR, in sorted brain endothelial cells from adult *Gpr124*$^{fl/+}$*; Cdh5-CreER, Gpr124*$^{fl/\Delta C}$*; Cdh5-CreER* and *Gpr124*$^{fl/-}$*; Cdh5-CreER* mice. Tamoxifen was administered into mice 4 doses every other day for 1 week, 3-4 weeks before experiments. Each value was normalized by *Gapdh* and littermate control *Gpr124*$^{fl/+}$*; Cdh5-CreER*. *Gpr124*$^{fl/+}$*; Cdh5-CreER*; n = 7, *Gpr124*$^{fl/\Delta C}$*; Cdh5-CreER* ; n=5, *Gpr124*$^{fl/-}$*; Cdh5-CreER*; n=5. Data represent mean ± SEM. Two-sided P-values were calculated by Tukey's multiple comparisons test. *p<0.05, **p<0.01, ***p<0.001, and ****p<0.0001.

Development • Supplementary information

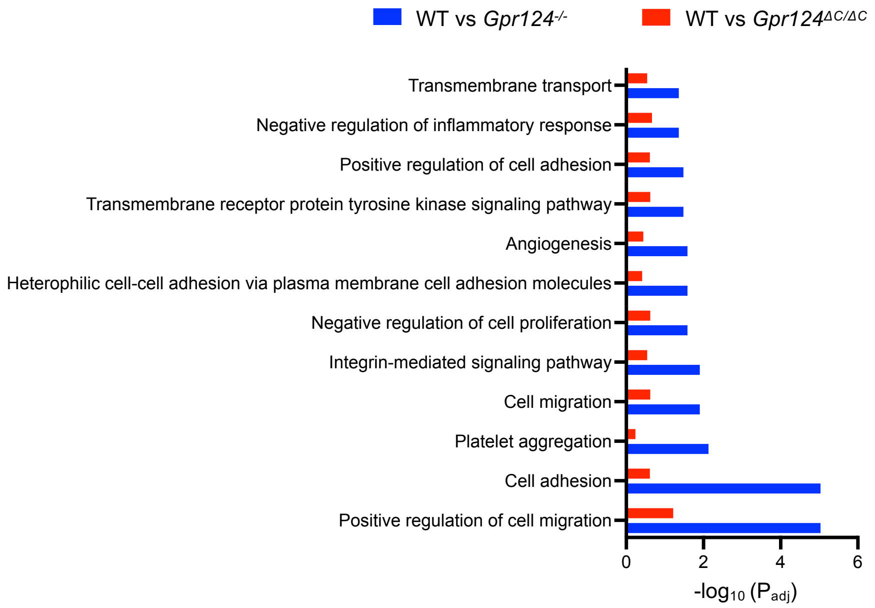

Fig. S7. Gene ontology enrichment analysis of differentially expressed genes in brain endothelial cells. GO terms enriched in WT vs *Gpr124*$^{-/-}$. The vertical axis represents GO categories, and the horizontal axis represents the -log10 (adjusted P-value) of WT vs *Gpr124*$^{-/-}$ and WT vs *Gpr124*$^{\Delta C/\Delta C}$. RNA-seq was performed with FACS-sorted brain EC cells from WT, *Gpr124*$^{\Delta C/\Delta C}$ and *Gpr124*$^{-/-}$ mice (n=4 mice per genotype), E15.5.

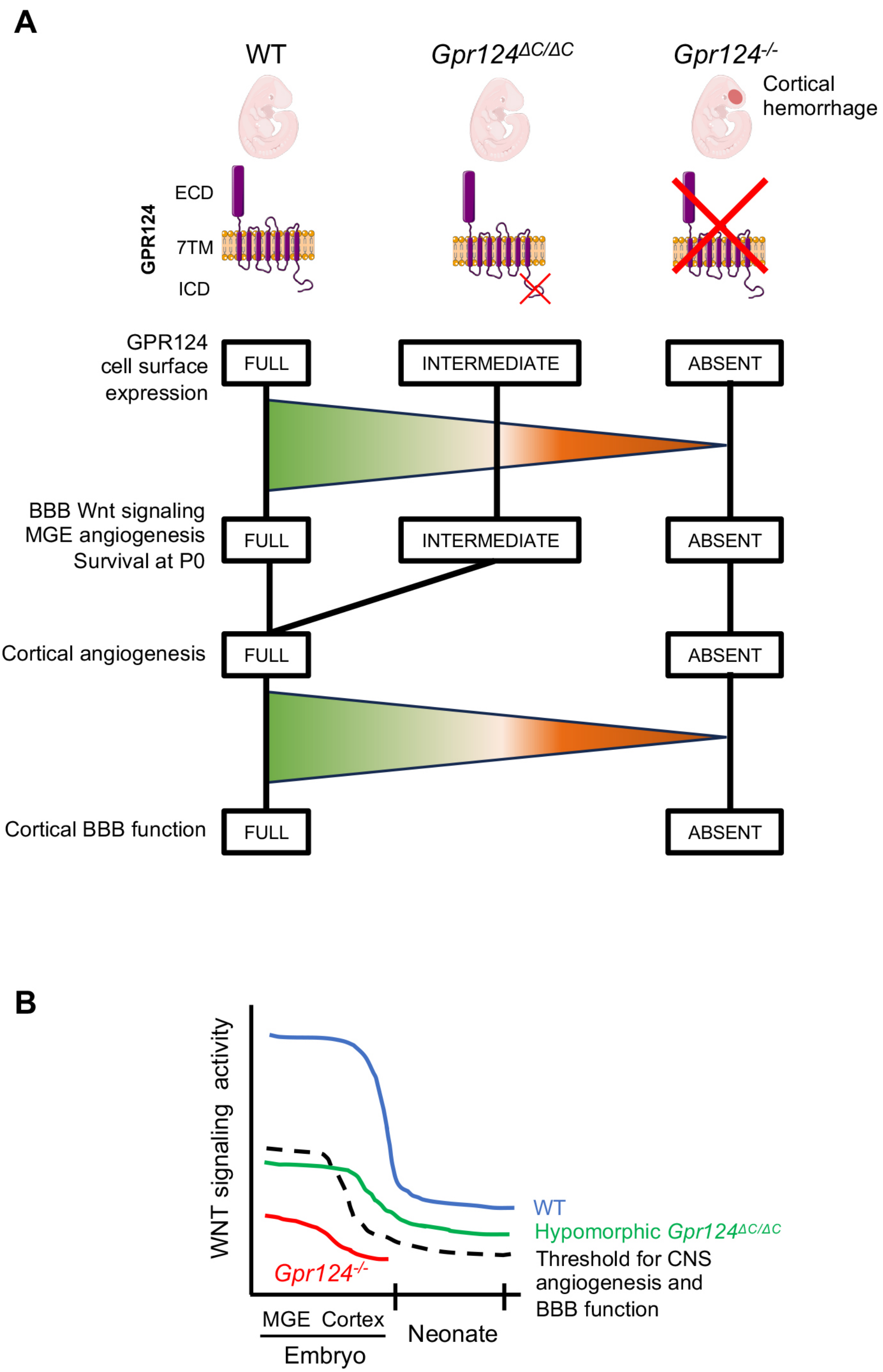

Development • Supplementary information

Fig. S8. Schematic graphics. (A) Summary of observed findings. **(B)** Representation of (A) depicting threshold level of BBB Wnt signaling required for cortical angiogenesis and BBB function, with exceeding of this threshold in *Gpr124*$^{\Delta C/\Delta C}$ mice but not *Gpr124*$^{-/-}$ *mice.* WNT signaling in brain endothelium is elevated during embryogenesis to promote CNS angiogenesis and BBB formation and then reduced to lower levels enough to maintain an intact BBB function. Brain regions have different sensitivity to the loss of WNT signaling activity. MGE is more sensitive than cortex. In hypomorphic *Gpr124*$^{\Delta C/\Delta C}$ mice, WNT signaling activity is decreased as compared to WT but higher than the level required to maintain CNS angiogenesis and an intact BBB function (threshold level), so CNS angiogenesis and BBB function are normal in the cortex. *Gpr124*$^{-/-}$ reduces WNT signaling much lower than the threshold level and this exhibits defective cortical CNS angiogenesis and BBB function. Based on our data and previous studies (Liebner et al., J Cell Biol 2008, Chang et al., Nature Medicine 2017, Cho et al., Neuron 2018). Figure was partly generated using Servier Medical Art, provided by Servier, licensed under a Creative Commons Attribution 3.0 unported license. ECD, extracellular domain; 7TM, 7-pass transmembrane domain; ICD, intracellular domain; BBB, blood- brain barrier.

Development • Supplementary information
